# Quantitative assessment of the probability of bluetongue virus overwintering by horizontal transmission: application to Germany

**DOI:** 10.1186/1297-9716-42-4

**Published:** 2011-01-11

**Authors:** Sebastian Napp, Simon Gubbins, Paolo Calistri, Alberto Allepuz, Anna Alba, Ignacio García-Bocanegra, Armando Giovannini, Jordi Casal

**Affiliations:** 1 Centre de Recerca en Sanitat Animal (CReSA), UAB-IRTA, Campus de la Universitat Autónoma de Barcelona, 08193 Bellaterra, Barcelona, Spain; 2 Institute for Animal Health, Pirbright Laboratory, Ash Road, Pirbright, Surrey, GU24 0NF, UK; 3 Istituto Zooprofilattico Sperimentale dell'Abruzzo e del Molise "G. Caporale", Via Campo Boario, 64100 Teramo, Italy; 4 Departament de Sanitat i Anatomia Animals, Universitat Autònoma de Barcelona, 08193 Bellaterra, Barcelona, Spain; 5 Departamento de Sanidad Animal. Facultad de Veterinaria, UCO, Campus Universitarios de Rabanales, 14071 Córdoba, Spain

## Abstract

Even though bluetongue virus (BTV) transmission is apparently interrupted during winter, bluetongue outbreaks often reappear in the next season (overwintering). Several mechanisms for BTV overwintering have been proposed, but to date, their relative importance remain unclear. In order to assess the probability of BTV overwintering by persistence in adult vectors, ruminants (through prolonged viraemia) or a combination of both, a quantitative risk assessment model was developed. Furthermore, the model allowed the role played by the residual number of vectors present during winter to be examined, and the effect of a proportion of *Culicoides *living inside buildings (endophilic behaviour) to be explored. The model was then applied to a real scenario: overwintering in Germany between 2006 and 2007. The results showed that the limited number of vectors active during winter seemed to allow the transmission of BTV during this period, and that while transmission was favoured by the endophilic behaviour of some *Culicoides*, its effect was limited. Even though transmission was possible, the likelihood of BTV overwintering by the mechanisms studied seemed too low to explain the observed re-emergence of the disease. Therefore, other overwintering mechanisms not considered in the model are likely to have played a significant role in BTV overwintering in Germany between 2006 and 2007.

## Introduction

Bluetongue (BT) is a non-contagious disease of ruminants, mainly sheep, caused by bluetongue virus (BTV), which belongs to the genus *Orbivirus *within the family *Reoviridae*. It is transmitted between hosts almost exclusively through the bites of the females of the *Culicoides *biting midge. BT is an OIE reportable disease and is of considerable socioeconomic concern and of major importance in the international trade of animals and animal products [1]. Before 1998, BT was considered an exotic disease in Europe with just a few sporadic incursions in the Iberian Peninsula. Between 1998 and 2005, different BTV strains affected several countries in the Mediterranean basin. In August 2006, BTV-8 was identified in the Netherlands, from where the disease spread to neighbouring countries. After a short winter break, BTV reappeared in 2007 causing a devastating epidemic [2]. Transmission of BTV is apparently interrupted during winter as a consequence of the low temperatures, which reduce the activity of vectors and BTV replication within them. However, once winter is finished, transmission often restarts [3]. A large number of mechanisms for BTV overwintering have been proposed.

Most *Culicoides *at northern latitudes survive the winter as larvae, and therefore the most logical explanation for overwintering was thought to be the vertical (transovarial) transmission of the virus from infected adult vectors to offspring [3]. However, even though viral RNA in larvae has been detected [4], the BTV itself could not be isolated. Persistence of BTV in the ruminant population may also occur by transmission between ruminants during sexual intercourse. Infected bulls may shed BTV in semen, but it seems to be restricted to old bulls and laboratory adapted viruses as there is no published report of isolation of BTV from semen of naturally infected bulls [5]. Recently, transmission of BTV-8 by direct contact, probably through ingestion of infected placentas, has been reported [6]. Vertical (transplacental) transmission of BTV has been described in both cattle and sheep, but was thought to be exclusively associated to cell-attenuated virus strains [7]. Nevertheless, in the case of BTV-8, transplacental transmission has been demonstrated both in the field [6,8-10] and experimentally [7], although, at least in naturally-infected sheep, its contribution to overwintering appears to be limited [11]. Besides, several other mechanisms for overwintering, none of which are yet sufficiently proven, have been proposed: (a) unidentified reservoir hosts [3], (b) alternative vectors such as ticks or biting flies [3], or (c) persistently infected ovine γδ T-cells [12].

However, before investigating all these particular overwintering mechanisms, it should first be clear how likely (ordinary) horizontal transmission could be responsible. This paper deals with the assessment of the probability of bluetongue virus overwintering by horizontal transmission. BTV may persist in the ruminant population during the winter, through a prolonged viraemia in some individuals. Infectious BTV can be isolated from the blood of cattle for much longer than from sheep and goats, and although the vast majority of infections in cattle endure for less than 60 days, a fraction may last for much longer [3]. Such infections could permit the virus to persist for months without infecting new hosts, and thereby survive short periods of vector absence. Besides, entomological surveillance systems in Northern Europe have demonstrated that small populations of *Culicoides *remain active during winter [13,14], and therefore year-round presence of adult infected *Culicoides *was considered as the most likely explanation for sustenance of the transmission cycle [15]. Nevertheless, BTV does not need to survive solely in either the host or the adult vector, but the mechanism for overwintering may be a combination of both. A *Culicoides *may infect the host before the end of the winter and the virus may reach the next season in the blood of infected ruminants (mainly cattle), when the conditions (presence of *Culicoides*) allow the re-emergence of disease.

The complete cessation of vector activity during winter, i.e. the vector free period (VFP), seems to be restricted to Afro-tropical species such as *C. imicola*, and only in specific areas of southern Europe. In other areas of Europe and with other *Culicoides *species, a period of total cessation of adult vector activity seems not occur. However, it is possible to identify periods of the year when the risk of transmission of BTV may be considered very low. This low transmission period (i.e. Period of Low Vector Activity; *PLVA*), will vary across Europe depending on the timing and duration of the local climate [15], and the biology of the vector species involved.

The assumption that *Culicoides *are purely exophilic (they will not enter or rest inside buildings) was attributed to the fact that most studies were performed in tropical areas or in the Mediterranean, on exophagic species like *C. imicola *[16]. However, studies in Northern Europe, have demonstrated that *Culicoides *are regularly found inside buildings [16-19] and that the endophagic behaviour appears to be driven primarily by external temperatures [16]. The ability of *Culicoides *to shelter from cold conditions inside farm buildings could extend the period of active BTV transmission [20], and that may have an impact on the probability of overwintering.

Therefore, the aim of the paper was to assess the probability of BTV overwintering by horizontal transmission by persistence of the virus in either adult vectors, ruminants (through prolonged viraemia) or a combination of both, by means of a stochastic risk assessment model. Besides, the model allowed assessing the role that the few *Culicoides *present during the *PLVA *and those which live inside buildings play on the probability of overwintering. The model was applied to a real scenario: overwintering in Germany between 2006 and 2007.

## Materials and methods

### Model pathways

**Figure 1:**
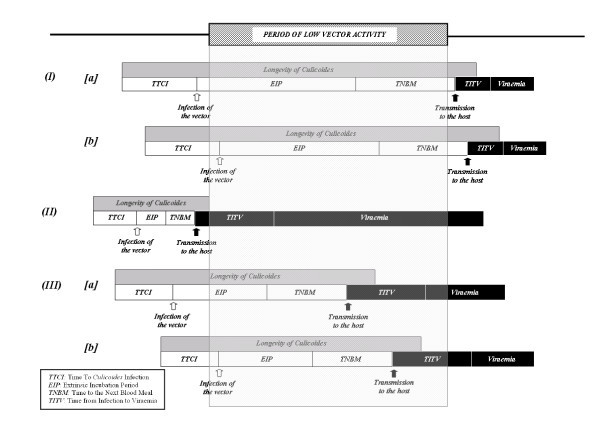
**Pathways for overwintering considered in the model: (I) horizontal transmission in the insect vectors, (II) horizontal transmission in the ruminant hosts and (III) horizontal transmission in the insect vector plus the ruminant population**. [a] represents infection of vectors before the *PLVA *and [b] infection of vectors during the *PLVA*. In pathways *Ia *and *IIIa*, the vectors need to have emerged before the PLVA, while in pathways *Ib *and *IIIb*, the vectors may have emerged before the PLVA, but also during the PLVA.

The model allowed the estimation of the probability of overwintering by different pathways (Figure 1):

*I*- Overwintering by long term persistence in the adult vector.

*II*- Overwintering by long term persistence in the ruminant host.

*III*- Overwintering by persistence in the vector plus the ruminant host.

In order to be able to transmit BTV, the vector needs to: (a) become infected (the number of days from the emergence of adult vectors to infection is called time to *Culicoides *infection (*TTCI*)), (b) be able to survive the extrinsic incubation period (*EIP*) and the time to the next blood meal (*TNBM*), and, (c) be able to effectively transmit BTV to a susceptible host. If the transmission to the host occurs beyond the *PLVA*, then overwintering was considered to have been achieved by persistence of BTV in the adult insect vectors (pathway *I*). If not, overwintering may still be achieved with the participation of the host. In this case, once the host becomes infected, there is a period until the animal becomes viraemic: time from infection to viraemia (*TIV*) and then a viraemic period. If the viraemic period goes beyond the end of the *PLVA*, then overwintering was considered to have been achieved by persistence of the virus in the adult vector plus the ruminant host (pathway *III*). If the host got infected before the start of the *PLVA *and the viraemic period went beyond the *PLVA*, then overwintering was considered to have been achieved by persistence of the virus in the ruminant hosts (pathway *II*).

In order to assess the role played by the small number of vectors present during the period of low vector activity, pathways *I *&*III *were further divided depending on whether the vectors were infected: [a] before the start of the *PLVA*, or, [b] during the *PLVA*.

Quantification of *Culicoides *population size is based on trapping, which samples only a proportion of the *Culicoides *population, so that the exact size of this portion is not known [18]. Consequently, the probabilities for each pathway (*Ia, Ib, IIIa *&*IIIb*) had to be estimated per vector. However, the model does allow quantification of the relative importance of these four different pathways. For pathway *II*, the overall probability may be estimated because the ruminant population in an area or country is usually known.

In order to explore the effect of a proportion of *Culicoides *living inside buildings and therefore subjected to a milder temperature during the winter months, the model was run (a) assuming exophilic behaviour exclusively and (b) assuming a proportion of vectors had endophilic behaviour (this proportion given by the probability of endophily on that month).

The model allows the estimation of these probabilities taking into account the specific conditions in a given country or area: (i) pattern of *Culicoides *activity throughout the year, (ii) temperatures, (iii) bluetongue incidence in both bovine and ovine in the previous season, and (iv) cattle and sheep populations.

### Risk assessment model

**Figure 2:**
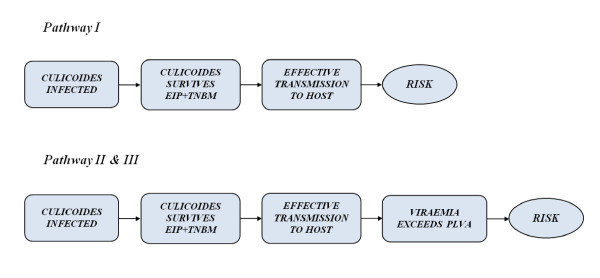
**Steps for overwintering for pathway *I *and pathways *II *and *III***.

For overwintering to occur, a series of events (steps) have to take place (Figure 2).

#### Probability of a Culicoides getting infected

Firstly, the probability of a *Culicoides *getting infected after a single blood meal was estimated as the product of: (1) the proportion of bites on cattle and sheep, (2) the probabilities of cattle and sheep being viraemic in month *i *(for *i *= November to April), and, (3) the proportion of bites on an infectious host that infect a midge.

Secondly, given a *Culicoides *which emerged on a given day, its longevity and the biting rate were calculated and used to estimate the number of blood meals the *Culicoides *had taken (*n*), which was then used to estimate the probability of infection after *n *blood meals.

#### Probability a Culicoides survives the *EIP *and the *TNBM*

Once the vector got infected, it needed to be able to survive the *EIP *(i.e. the time from the ingestion of the virus until it reaches the salivary glands) and the *TNBM*, so that BTV can be transmitted to a susceptible host.

#### Probability of effective transmission

Probability of effective transmission was estimated taking into account: (1) the proportion of bites on cattle and on sheep, (2) the proportion of cattle and sheep which are susceptible (not immune), and (3) the proportion of bites per infectious midge that infect a host.

#### Probability the viraemia goes beyond the end of the PLVA (for pathways II and III)

This probability was estimated taking into account: (1) the time from infection to viraemia, and (2) the duration of viraemia in cattle or sheep.

A detailed explanation of the model calculations for the different steps is available in additional file 1.

### Expert opinion workshop

Some parameters for which quantitative data were not available were estimated based on the opinion of experts. The method employed to elicit the opinion of experts was the Workshop Method, and was carried out during the First MedReoNet Annual meeting held in Palma of Majorca (Spain).

### Modelling software

The spreadsheet model was constructed in Microsoft Excel (Microsoft^® ^Office Professional Edition, 2003), and run for 50 000 iterations (Latin Hypercube sampling) in @Risk version 4.5.5 (^© ^Palisade Corporation).

### Sensitivity analysis

In order to identify those input parameters which were more influential in the model output(s), a sensitivity analysis was carried out. For each month, a regression analysis (either linear or logistic regression) was performed independently for the different steps in the transmission pathway: (1) Probability *Culicoides *getting infected, (2) Probability *Culicoides *survives *EIP *and *TNBM*, and (3) Probability of effective transmission. Furthermore, a second regression analysis to assess the influence of these steps in the final weighted probability was carried out. For these analyses, the results of each iteration of (i) those input parameters which influenced these different steps (Table 1), (ii) the probabilities associated to these steps, and also (iii) the final weighted probability, were extracted from the model.

**Table 1:** Input parameters included in the sensitivity analysis of the different outputs

Outputs (Steps)	Inputs
Probability of *Culicoides *infection (per month)	Proportion of bites on cattle and on sheep Within farm prevalence in cattle Within farm prevalence in sheep Probability of viraemia month 0 to 3 in cattle and sheep Proportion of bites on infectious host that infect a midge Proportion of bites per infectious midge that infect a host Longevity of *Culicoides *(per month) Mean number of blood meals (per month)
Probability of surviving the *EIP *and the *TNBM *(per month)	Longevity of *Culicoides *(per month) Extrinsic Incubation Period (per month) Time to the Next Blood Meal (per month)
Probability of effective transmission	Proportion of bites on cattle and on sheep Proportion of bites per infectious midge that infect a host

For quantitative outcomes, the relative strength of the input parameters was measured by the value of the standardized coefficient (beta). For categorical dichotomous outcomes, the relative strength of the input parameters was measured by the values of the Wald estimate and the *exp(B)*.

The analyses were performed using SPSS 17.0.0 (Statistical Package for Social Sciences Inc., Chicago, IL, USA). A more detailed explanation of the sensitivity analysis is available in additional file 1.

### Scenario description

The model was applied to a real scenario: overwintering in Germany in 2006-2007. In 2006, BTV-8 was detected in Germany affecting 571 cattle farms and 309 sheep flocks. The region affected was mainly North Rhine-Westphalia, nearby the affected areas in Belgium, the Netherlands, and Luxembourg. Apparently, the infection overwintered in the region, and in 2007 spread over most of Germany [21].

The specific inputs for the German scenario are shown in Table 2.

**Table 2:** Specific input parameters (Germany 2006-2007)

Description of model input parameter	Value		Source
Mean daily temperatures (°C)	Various (see Figure 3)		^1^
Monthly proportion of *Culicoides *captures during study period (November to April)	Nov.: 0.977 Dec.: 0.017 Jan.: 0.002	Feb.: 0.001 Mar.: 0.001 Apr.: 0.001	[5]
Monthly proportion of *Culicoides *captured outdoors (versus indoors)	Nov.: 0.50 Dec.: 0.40 Jan.: 0.27	Feb.: 0.12 Mar.: 0.32 Apr.: 0.17	[5]
Cattle population in North Rhine-Westphalia (*H_c_*)	1 346 488		^2^
Sheep population in North Rhine-Westphalia (*H_s_*)	199 762		^2^
Monthly cumulative incidence of cattle farms (*CI_ci_*)	Aug. 2006: 1.8 × 10^-3^ Sep. 2006: 3.0 × 10^-3^ Oct. 2006: 1.4 × 10^-2^ Nov. 2006: 8.5 × 10^-3^ Dec. 2006: 2.0 × 10^-3^	Jan. 2007: 4.2 × 10^-3^ Feb.2007: 2.7 × 10^-3^ Mar. 2007: 1.0 × 10^-3^ Apr. 2007: 1.6 × 10^-3^	^2, 3^
Monthly cumulative incidence of sheep farms (*CI_si_*)	Sep. 2006: 1.1 × 10^-2^ Oct. 2006: 4.6 × 10^-2^ Nov. 2006: 2.5 × 10^-2^ Dec. 2006: 4.0 × 10^-3^	Jan. 2007: 0 Feb. 2007: 0 Mar. 2007: 0 Apr. 2007: 0	^2, 3^
Proportion of immune cattle	0.01		Model estimation^‡^
Proportion of immune sheep	0.04		Model estimation^‡^

^1^Anonymous: Bundesministerium für Verkher, Bau und Stadtentwicklung. Klimadaten Deutschland. http://www.dwd.de/bvbw/appmanager/bvbw/dwdwwwDesktop? [consulted 6 August 2009]

^2^Anonymous: Statische Ämter des Bundes und der Länder. https://www.regionalstatistik.de/ [consulted 6 August 2009]

^3^Anonymous: EU. Food Safety Regulatory Committees: Standing Committee on the Food Chain and Animal Health (SCFCAH). http://ec.europa.eu/food/committees/regulatory/scfcah/animal_health/presentations_en.htm#03042009 [consulted 8 August 2009]

^‡ ^The proportion of immune cattle and sheep were obtained based on the estimated number of cattle and sheep infected in 2006 (natural immunity) as vaccination did not start until 2008

**Figure 3:**
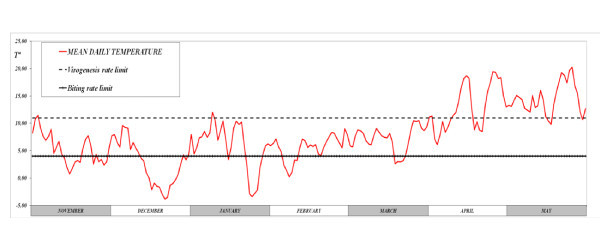
**Mean daily temperatures (red line) for November to May in North Rhine-Westphalia**. Virogenesis rate limit (blue line) and biting rate limit (green line). Source: Bundesministerium für Verkher, Bau und Stadtentwicklung. Klimadaten Deutschland. http://www.dwd.de/bvbw/appmanager/bvbw/dwdwwwDesktop?

Based on *Culicoides *catches in Germany a *PLVA *of four months (between January and April) was considered. The two months previous to the *PLVA *(November and December) were also considered for the analysis. The probabilities of overwintering by *Culicoides *emerged in each of these months were estimated. The mean daily temperatures in the area of study for the months considered (plus May) are represented in Figure 3.

The relative importance of the different pathways (*I, II *and *III*), and of overwintering by vectors infected before the start of the *PLVA *[a] or vectors infected during the *PLVA *[b], were assessed. Furthermore, the importance of the endophilic behaviour of *Culicoides *was also assessed by comparing the results (i) assuming that all the vectors were subjected to the outside temperatures, and (ii) assuming that the vectors had a certain probability of being inside, and therefore subjected to the inside temperatures. These probabilities were given by monthly proportion of *Culicoides *captured indoors versus outdoors (Table 2). The temperatures inside buildings were assumed not to vary widely because most of buildings in Northern Europe are likely to be closed, and the presence of animals contributes to the maintenance of the heat. Therefore, when outside temperatures were below 0°C, inside temperatures were supposed to range between 10 and 15°C, while when outside temperatures were above 0°C, inside temperatures were supposed to range between 15 and 20°C.

## Results

The results are presented in two forms (Table 3):

**Table 3:** Results: Mean probabilities per vector for the different pathways and months of emergence of *Culicoides *given exophilic and endophilic behaviour.

Results per vector	Mean probability *Ia*		Mean probability *Ib*		Mean probability *IIIa*		Mean probability *IIIb*		Mean probability (per month)	
	Exophilic	Endophilic	Exophilic	Endophilic	Exophilic	Endophilic	Exophilic	Endophilic	Exophilic	Endophilic
November	0	0	0	0	0	0	0	0	0	0
December	0	0	0	0	0	0	0	0	0	0
January	NA	NA	0	0	NA	NA	0	1.2 × 10^-8^	0	1.2 × 10^-8^
February	NA	NA	5.9 × 10^-8^	5.5 × 10^-8^	NA	NA	0	6.7 × 10^-8^	5.9 × 10^-8^	1.2 × 10^-7^
March	NA	NA	9.2 × 10^-8^	8.7 × 10^-8^	NA	NA	0	2.1 × 10^-7^	9.2 × 10^-8^	3.0 × 10^-7^
April	NA	NA	1.1 × 10^-7^	1.6 × 10^-7^	NA	NA	0	5.1 × 10^-9^	1.1 × 10^-7^	1.6 × 10^-7^

Weighted-results	Probability Ia		Probability Ib		Probability IIIa		Probability IIIb		Total months	
	Exophilic	Endophilic	Exophilic	Endophilic	Exophilic	Endophilic	Exophilic	Endophilic	Exophilic	Endophilic

November	0	0	0	0	0	0	0	0	0	0
December	0	0	0	0	0	0	0	0	0	0
January	NA	NA	0	0	NA	NA	0	1.4 × 10^-9^	0	1.4 × 10^-9^
February	NA	NA	1.6 × 10^-10^	6.2 × 10^-11^	NA	NA	0	1.3 × 10^-9^	1.6 × 10^-10^	1.3 × 10^-9^
March	NA	NA	1.2 × 10^-9^	1.6 × 10^-9^	NA	NA	0	3.6 × 10^-9^	1.2 × 10^-9^	5.1 × 10^-9^
April	NA	NA	9.4 × 10^-9^	2.3 × 10^-8^	NA	NA	0	1.8 × 10^-9^	9.4 × 10^-9^	2.5 × 10^-8^
**Mean probability (per pathway)**	**0**	**0**	**1.1 × 10^-8^**	**2.4 × 10^-8^**	**0**	**0**	**0**	**8.0 × 10^-9^**	**1.1 × 10^-8^**	**3.2 × 10^-8^**

Weighted mean probabilities for the different pathways and months of emergence of *Culicoides *given exophilic and endophilic behaviour. Mean probabilities for the different months for pathway *II *were zero, and therefore are not shown in the table.

NA: Not applicable (in pathways *Ia *and *IIIa *the vectors have to get infected before the start of the PLVA and therefore only apply to vectors emerged before the start of the PLVA, i.e. December)

- Per vector, i.e. given a vector which emerges in a given month, we estimated the probability it resulted in overwintering by each of the pathways considered. Results are presented both assuming exophilic behaviour exclusively and assuming that a proportion of vectors had endophilic behaviour.

- Weighted by the proportion of vectors which emerge in that month out of the total *Culicoides *emerged throughout the period of study. Differences were also made between exophilic behaviour exclusively and assuming that a proportion of vectors had endophilic behaviour.

The results per vector (Table 3) indicate that for exophilic *Culicoides *overwintering was only possible by vectors infected during the *PLVA *that infected the host after this period is finished (pathway *Ib*), and only by vectors that emerged after January, with the mean probabilities increasing between February (5.9 × 10^-8^) and April (1.1 × 10^-7^). Endophilic behaviour allowed transmission by both vectors infected during the *PLVA *that infect the host after this period is finished (pathway *Ib*) and by vectors infected during the *PLVA *that infect the host before this period is finished (pathway *IIIb*). This allowed advancing the period in which transmission was possible (to January). The mean probabilities of overwintering increased between January (1.2 × 10^-8^) and April (1.6 × 10^-7^).

Overwintering by long term persistence in the ruminant host (pathway *II*) was not possible.

Of the steps considered in the pathways for overwintering (Figure 2), the main determinants of the low probabilities obtained were the low likelihood of *Culicoides *infection and the low probability of *Culicoides *surviving the *EIP *and the *TNBM*. The probabilities of *Culicoides *infection for the different months were consistently higher for endophilic *Culicoides *as compared to exophilic (Table 4), although the differences decreased gradually. Similarly, endophilic behaviour increased the probabilities of surviving the *EIP *and the *TNBM *(Table 4). The probabilities of effective transmission were always in the range of 0.9 and therefore did not have a great influence in the final result.

**Table 4:** Probabilities of *Culicoides *infection and probabilities of *Culicoides *surviving the *EIP *and *TNBM *for exophilic and endophilic *Culicoides *per month of emergence

	Mean probability *Culicoides *infected		Mean probability *Culicoides *survives *EIP *+ *TNBM*	
	
	Exophilic *Culicoides*	Endophilic *Culicoides*	Exophilic *Culicoides*	Endophilic *Culicoides*
November	4.1 × 10^-5^	1.4 × 10^-4^	0	1.4 × 10^-3^
December	8.9 × 10^-6^	4.0 × 10^-5^	0	1.6 × 10^-4^
January	1.4 × 10^-5^	2.6 × 10^-5^	0	2.4 × 10^-4^
February	2.6 × 10^-5^	4.1 × 10^-5^	5.4 × 10^-4^	1.8 × 10^-3^
March	2.3 × 10^-5^	2.8 × 10^-5^	7.8 × 10^-4^	2.3 × 10^-3^
April	2.0 × 10^-5^	2.0 × 10^-5^	2.0 × 10^-5^	2.0 × 10^-5^

The sensitivity analysis showed that, for both the exophilic and endophilic scenarios, the most influential parameters in the probability of infection for the different months were the total number of blood meals, with mean values of the standardized coefficient (beta) of 0.57 and 0.68 for the exophilic and endophilic scenarios respectively; and the proportion of bites per infectious midge that infect a host, with mean values of beta of 0.37 and 0.31 for the exophilic and endophilic scenarios respectively. The longevity of *Culicoides *was eliminated from the regression model because of its statistically significant correlation to the number of blood meals, which was weaker in the case of endophilic *Culicoides*. For the probability of *Culicoides *surviving the *EIP *and the *TNBM*, the longevity of *Culicoides *was the most influential parameter (mean value of Wald statistic for both scenarios of 212). The values of *exp(B)*, that give the odds ratios, indicated that the longer a *Culicoides *live, the higher the probability it survives the *EIP *and the *TNBM*, although this increase was higher for exophilic *Culicoides *(mean *exp(B) *of 1.2 as compared to 1.1 for endophilic *Culicoides*). *TNBM *was also statistically significant, but the values of the Wald tests were much lower (mean value of 23 for both scenarios). The pattern of values of *exp(B) *is less clear, in general the shorter the *TNBM*, the higher the probability the *Culicoides *survives the *EIP *and the *TNBM*, but for some months in the exophilic scenario, the effect seemed to be the opposite. The *EIP *had to be eliminated from the regression model because of its statistically significant correlation with longevity. The only exception was for April in the endophilic scenario. The value of *exp(B) *indicated that the lower the *EIP*, the higher the probability the *Culicoides *survives the *EIP *and the *TNBM*. The most influential parameters in the probability of effective transmission was the proportion of bites per infectious midge that infect a host (beta = 0.86), while the proportion of bites on cattle and on sheep (beta = 0.51) seemed less important.

For exophilic *Culicoides *the mean weighted result (Table 3) was 1.1 × 10^-8^, and almost 90% of the risk of overwintering was due to *Culicoides *emerged in April. For endophilic *Culicoides *the mean weighted results (Table 3), and a 78% of the risk was due to *Culicoides *emerged in April.

The assessment of the influence of the different steps in the final weighted probability indicated that by far the most influential step was the probability that *Culicoides *emerged in April survived the *EIP & TNBM *(beta = 0.34 and 0.40 for exophilic and endophilic *Culicoides *respectively). The second most influential step was that *Culicoides *emerged in March survived the *EIP *and *TNBM *(beta = 0.06 and 0.08 for exophilic and endophilic *Culicoides *respectively). The probability of infection of the *Culicoides *emerged in April was the third most determinant parameter (beta = 0.02 and 0.04 for exophilic and endophilic *Culicoides *respectively).

## Discussion

In Germany, between 2006 and 2007, the length of the *PLVA *(4 months) did not allow overwintering by midges emerged before this period (pathways *Ia *and *IIIa*) neither with the exophilic nor with the endophilic behaviour. This long *PLVA *did not allow overwintering by hosts infected before the *PLVA *(pathway *II*) either.

For exophilic *Culicoides*, overwintering was only possible by pathway *Ib *as temperatures above the virogenesis rate limit were reached only a few days in April (Figure 3), which did not allow the completion of the *EIP *and *TNBM*, and transmission to the host before the end of the *PLVA *(pathway *IIIb*). Endophilic behaviour appeared to favour overwintering mainly by increasing the probability by pathway *Ib*, and to a lesser extent by allowing the transmission of BTV to ruminants during the *PLVA *(pathway *IIIb*), which allowed advancing the period in which transmission was possible (to January). In fact, mild temperatures inside buildings did allow vectors emerged throughout the whole study period to survive the *EIP *and the *TNBM*. However, for vectors emerged in November and December, the duration of the *PLVA *(4 months) did not allow infected vectors (pathway *Ia*), or viraemic hosts (pathway *IIIa*) to reach May.

Overall, the sensitivity analysis highlighted the importance of the temperature-dependent parameters (longevity, *EIP *and *TNBM*) on the probability of BTV overwintering, although their relative importance is difficult to assess because of the correlation that exists among these parameters. The importance of longevity may be understood because of its influence in both the probability of infection and the probability of surviving the *EIP *and the *TNBM*. On the other hand, the duration of the *TNBM *seemed to have a less decisive role in the probability of overwintering, which might be explained by the fact that when temperatures were favourable for the completion of the *EIP*, they also allowed the rapid completion of the *TNBM*.

Of the non temperature-dependent parameters, the proportion of bites on an infectious host that infect a midge seemed to be the most influential. There is a great degree of uncertainty regarding this parameter as the distribution used was a combination of field estimates *C. sonorensis *and laboratory estimates for *C. obsoletus*, and variations in viral titres within the host and among different hosts, were not taken into account.

The results of the sensitivity analysis are in agreement with previous studies [22], and emphasize the need for further research in the estimation of these influential parameters.

Even though endophily seemed to favour overwintering, its effect was limited (the mean weighted probabilities were less than three times higher than for exophilic *Culicoides*). This is a consequence of the complex effect of temperature on BTV transmission: an increase of temperature reduces the duration of the *EIP *and the *TNBM*, but also the longevity of *Culicoides*; and a decrease of temperature increases the longevity of *Culicoides*, but also the duration of the *EIP *and the *TNBM*. Therefore, even though endophily (milder temperatures) increased the probability of vector infection (Table 4), this probability is the result of the equilibrium between longevity and number of blood meals, and while endophily increased the number of blood meals in relation to exophily (lower temperatures), it also decreased longevity. Similarly, endophily increased the probability of surviving the *EIP *and the *TNBM *(Table 4), but again, this probability is the result of the equilibrium between longevity and duration of the *EIP *and the *TNBM*, and while endophily decreased the duration of these two periods in relation to exophily, it also decreased longevity. This is somehow no unexpected because it is known that BTV transmission by *Culicoides *is inefficient, and that very few ever transmit the virus, so this has to be compensated by huge numbers of vectors [23]. Given the low probabilities obtained for the pathways considered in the model, for these mechanisms to have played a major role in overwintering in Germany, the number of vectors present in winter would have had to be large. Even though *Culicoides *captured represent only a fraction of the *Culicoides *population, the number of *Culicoides *trapped during winter in Germany seems too small (captures during the *PLVA *represent only a 0.06% of the total of the year).

The low probabilities are consistent with what was observed in northern Europe, where the disease reappeared around areas of intense transmission rather than those where the transmission was most recent [15], and nearly all the northern European countries previously infected [18]. In fact, BTV isolation from overwintering populations of *Culicoides *has not been achieved yet [15]. Therefore, other overwintering mechanisms not considered in the model seem to have played a decisive role in overwintering in Germany. In 2008, transplacental transmission of field strains of BTV-8 was demonstrated in Northern Ireland [6]. Before this, it was thought only viruses passaged in tissue culture had the potential to cross the placenta, but since then, similar findings have been reported in several European countries [8-10]. However, whether PCR positive calves born to dams naturally infected during pregnancy are able to infect midges, and therefore play a role in overwintering is unknown [8,10]. Besides, mechanisms considered of minor significance during normal transmission, may become disproportionately important for the survival of the virus when normal transmission is interrupted by winter, and one or more of these mechanisms may be responsible for the cases of BTV transmission that have taken place during the winter in NW Europe [2].

The model was applied to a given scenario, in this case Germany in 2006-2007 taking into account its specific conditions. Therefore, any conclusions drawn are specific of that scenario as different conditions (e.g. temperatures or duration of *PLVA*) may produce different results. In addition, different *Culicoides *species may differ in their ability to transmit BTV [22,24]. However, given the lack of species-specific data, all suspect and confirmed vector species were considered equally competent in transmitting all BTV serotypes, as recommended by EFSA [15]. In the proposed scenario (Germany), this is unlikely to have played a decisive role as *Culicoides obsoletus *was by far the most common species accounting for at least 70% of total captures, and more than 90% on some farms [25].

Only sheep and cattle were considered in the model. Even though goats are also susceptible to BTV, in the case of Germany, given the low number of goats, they are unlikely to have played an important role in BTV transmission. In fact, they constituted only a 0.35% of the infected domestic ruminants reported in Germany in 2007 [21]. In countries with larger goat populations (e.g. Southern European countries), they may need to be taken into account. Several species of wild ruminants are known to be susceptible to BTV infection, and in Germany BTV-8 has been detected in red deer, fallow deer, roe deer and mouflon [21]. However, the role played by these species on the epidemiology of BTV in Europe is difficult to predict. Other factors besides temperature, such as humidity may affect the transmission of BTV, as shown by Wittmann et al. [26], but they were not taken into account because of the lack of data on the effect of humidity at different temperatures.

Besides, both variable and uncertain parameters were used, and that constrains the assessment of the relative contribution of variability and uncertainty on the results.

One advantage of the model is that it allows the estimation of the probabilities taking into account the specific conditions in a given country or area: (i) pattern of *Culicoides *activity throughout the year, (ii) bluetongue incidence in both bovine and ovine in the previous year, and (iii) cattle and sheep populations. Furthermore, the model allows taking into account the effect of temperature in BTV transmission. Vectors are not maintained at constant temperatures and therefore the effect of daily variations in temperatures needs to be considered. In fact, it has been observed that in cool conditions orbiviruses may persist in vectors for long periods, and that subsequent exposure to warm temperatures resulted in replication of this latent virus allowing transmission [26].

The model provides a framework which may be useful for the assessment of the probability of overwintering of other vector-borne diseases, in particular other orbiviruses such as Epizootic Hemorrhagic Disease (EHD) or African Horse Sickness (AHS).

## Competing interests

The authors declare that they have no competing interests.

## Authors' contributions

SN conceived of the study, development of the model, performed model calculations and drafted the manuscript. SG participated in the design of the model. PC participated in the design of the model. AA participated in the design of the model. AA participated in the design of the model. IGB participated in the design of the model. AG participated in the design of the sensitivity analysis. JC conceived of the study, and participated in its design and coordination. All authors read and approved the final manuscript.

## Supplementary Material

Additional file 1 **Model calculations**. The file contains a detailed explanation of the model calculations for the different steps. Click here for file
