# Supplementary material for: Quantitative assessment of the probability of bluetongue virus overwintering by horizontal transmission: application to Germany
Source: Vet Res. 2011 Jan 11;42(1):4. doi: 10.1186/1297-9716-42-4 (PMC3031226; doi:10.1186/1297-9716-42-4)
Supplement: Additional file 1 — Model calculations. The file contains a detailed explanation of the model calculations for the different steps. [file 1297-9716-42-4-S1.DOC]

**SUPPLEMENTARY MATERIAL**

**1. Risk assessment model**

Those inputs (i.e. parameters that are fed to the model) which are general are presented in Table S1. The outputs (i.e. parameters obtained by model calculations) which are general are presented in Table S2. Input parameters specific of the German scenario are presented in Table 2.

For overwintering to occur, a series of events (steps) have to take place (Figure S1).

***1.1. Probability of a Culicoides getting infected***

1.1.1 Firstly, the probability of a *Culicoides* getting infected after one blood meal for month *i* (*P_Ii_*) was estimated as the product of: the proportions of bites on cattle and sheep (*Φ_c_* and *Φ_s_*, respectively); the monthly probabilities of cattle and sheep being viraemic (*P_Vci_* and *P_Vsi_*, respectively); and the proportion of bites on an infectious host that infect a midge (*α*).

1.1.1.1. Proportion of bites on cattle and sheep

The proportions of bites on cattle (*Φ_c_*) and on sheep (*Φ_s_*) were calculated as [1]:

$\varphi_{c}=H_{c}/(H_{c}+\sigma H_{s});\varphi_{s}=1-\varphi_{c}$

Where *H_c_* and *H_s_* represented the cattle and sheep population in the affected area respectively (Table 2), and *σ* was a measure of the vector preference for cattle compared to sheep (if *σ* *< 1*, the vectors feed preferentially on cattle, and if *σ > 1*, the vectors feed preferentially on sheep). A hypothetical scenario of a farm with 10 cattle and 10 sheep, was used to obtain from the experts, an estimate of the proportion of vectors biting on cattle: *Φ_c10_* (Table S1), which was then used to calculate the value of *σ*.

1.1.1.2. Probabilities of cattle and sheep being infectious (viraemic) in month *i* (*P_Vci_* and *P_Vsi_* respectively), for *i*= November to April

First, the probability of a cow being viraemic in month *i* given infection in that same month (*i*): *p_vci_* was calculated as:

*p_vci_ = d_vci_*/ *30*

Where *30* represented the mean duration of a month in days, and *d_vci_* represented the days the cow remained viraemic in month *i*, which was calculated as:

*d_vci_* = *30 – (DI+ TIV_c_)*

For: *0 ≤ d_vci_ ≤ D_Vc_*

Where *DI* represented the day of infection within a month, which was modelled by a *Uniform (1; 30)* distribution; *TIV_c_* the time between infection and viraemia for cattle (Table S1); and *D_Vc_* the duration of viraemia for cattle (Table S1).

Similarly, the probability of a cow being viraemic in month *i+1* given infection in month *i* (*p_vci+1_*) was calculated as:

*p_vci+1_ = d_vci+1_/ 30*

Where *d_vci+1_* represented the days the cow remained viraemic in month *i+1*, which was calculated as:

*d_vci+1_* = *D_Vc_* – *d_vc_*_i_

For: *0 ≤ d_vci+1_ ≤ 30*

Likewise, the probabilities for months *i+2,…* were also calculated.

Monte Carlo simulations were used for the calculation of the probabilities (*p_vci_*, *p_vci+1_*,..), and the results were used to construct empirical (non-parametric) cumulative distributions of the probabilities of a cow being viraemic in the different months after infection, which were used as inputs of the model. The mean values of these distributions are shown in Table S1.

Then, the probabilities of a cow being viraemic for the months included within the study period (November to April: *P_VcNov_* to *P_VcApr_*) were calculated taking into account the probabilities of a cow being viraemic in month *i, i+1,..* given infection in month *i* (*p_vci_*, *p_vci+1_*,..) and the probabilities of a cow being infected in months of August to April (as a cow infected in August may still be viraemic in November).

The probability of a cow being infected in month *i* (*ρ_i_*) was calculated as:

$\rho_{ci}=CI_{ci}\times\omega_{c}$

Where *CI_ci_* was the cumulative incidence of cattle farms in month *i* (Table 2), and *ω_c_* the within farm incidence in cattle.

Similarly, using the specific inputs for sheep, the probabilities of a sheep being viraemic in the different months of the year (*P_Vsi_*) were also calculated.

The probabilities of cattle and sheep being viraemic in November and December will determine the probabilities of infection of the vectors for pathways *Ia* and *IIIa*, while the probabilities of cattle and sheep being viraemic in January to April will determine the probabilities of infection of the vectors for pathways *Ib* and *IIIb* (Figure 1).

1.1.1.3. Proportion of bites on infectious host that infect a midge: *α* (Table S1)

Finally, the probability of a *Culicoides* getting infected after one blood meal for month *i* (*P_Ii_*) was calculated as:

$P_{Ii}=\left[\varphi_{c}\times P_{Vci}\times\alpha\right]+\left[\varphi_{s}\times P_{Vsi}\times\alpha\right]$

1.1.2. Probability *Culicoides* getting infected after *n* blood meals (*P_In_*)

The longevity of *Culicoides* and the biting rate determine the number of blood meals the vector has taken and therefore its probability of infection.

1.1.2.1. Longevity of *Culicoides*

The mortality rate of *Culicoides* (*m_r_*) depending on the temperature (*T*) was calculated as [1, 2]:

$m_{r}(T)=0.009\times\exp(0.16\times T)$

And the daily probability of survival (*P_S_*) was calculated as:

$P_{S}=\exp(-m_{r})$

The vectors are not maintained at a constant temperature, and therefore mean daily temperature data was used to calculate the daily mortality rates and the daily probabilities of survival for the different days of the year.

The day of the year an adult *Culicoides* emerges will determine the values of all the temperature-dependent parameters which affect BTV transmission. Therefore, the probability of an adult midge emerging in each particular day of the year had to be estimated from the proportion of *Culicoides* trapped each month. In order to do that, the days from emergence to capture for month *i* (modelled by a *Uniform (1; d_i_)* distribution, where *d_i_* represented the mean longevity for month *i*), was subtracted from the day of capture within month *i* (modelled by a *Uniform (1; 30)* distribution), to estimate the proportions of the *Culicoides* trapped in month *i* that had emerged in month *i*, month *i-1*,…

Then, given a *Culicoides* which emerged in a particular day of the year (*x*), the probability that it survives just one day (i.e. until *x+1*): *P_Sx1_*, was calculated as:

$P_{Sx1}=(P_{Sx})\times(1-P_{Sx+1})$

And the calculations of the probabilities associated to the survival of different number days are presented in Table S3. These values of days of survival and associated probabilities were used to construct a discrete distribution, which represents the longevity of the *Culicoides* emerged on day *x*. Based on [3], the maximum *Culicoides* longevity was set at 120 days. To account for the effect of low temperatures, when *Culicoides* were subjected to temperatures under 0 ºC for 3 days within a period of 10 days, they were assumed to die (R. Meiswinkel personal communication based on experience in the field).

1.1.2.2. Biting rate

The *Culicoides* biting rate as a function of temperature: *b_r_*(*T*) may be calculated as:

$b_{r}(T)=0.000171\times T\times(T-3.6966)\times(41.8699-T)^{1/2.7056}$

The equation is only valid for temperatures above 4 ºC (for temperatures below this value, transmission was assumed to stop).

Mean daily temperature data allowed the calculation of the biting rates for the different days of the year. Then, the daily probability the vector has taken a blood meal on day *x* (*P_BMx_*) was calculated as:

$P_{BMx}=1-\exp(-b_{rx})$

Where *b_rx_* represented the biting rate for day *x*.

The probability of a *Culicoides* getting infected on day *x* (*P_Ix_*) was calculated as:

$P_{Ix}=P_{Ii}\times P_{BMx}$

Where *P_Ii_* was the probability of a *Culicoides* getting infected after 1 blood meal in month *i* (the month to which day *x* belongs).

Finally, for a *Culicoides* emerged on day *x*, and whose longevity is given by *d* days, the probability that the vector getting infected by BTV (*P_In_*) was calculated as:

$$P_{In}=1-\prod_{j=0}^{d} (1-P_{Ix+j})$$

1.1.2.3. Time to *Culicoides* infection (*TTCI*)

The *TTCI* in a given iteration (*r*) is obtained from:

$r=Discrete\left(k;p_{k}\right)$

Where *k* represents the days in which a *Culicoides* may get infected, and goes from 1 to *d* (longevity of the *Culicoides*) days; and *p_k_* the probability of infection on day *k*. The values of the Discrete distribution for the calculation of the *r* of a *Culicoides* which emerged on day *x* are shown in Table S4.

***1.2. Probability a Culicoides survives the extrinsic incubation period (EIP) and the time to the next blood meal (TNBM)***

In order to transmit the disease, the vector, once infected, needs to be able to survive the Extrinsic Incubation Period (*EIP*) and the Time to the Next Blood Meal (*TNBM*).

1.2.1. Extrinsic Incubation Period (*EIP*)

The virogenesis rate (*v_r_*) depending on the temperature (*T*) may be estimated as [4]:

$v_{r}(T)=0.0003\times T\times(T-10.4057)$

The equation is only valid for temperatures above 11ºC, while for temperatures below this value, the virogenesis was assumed to stop.

The reciprocal of the virogenesis rate is the *EIP*, defined as the time between the infection of the vector and when it first becomes capable of transmitting the virus, was calculated as:

$EIP(T)=1/v_{r}(T)$

Mean daily temperature data was used to calculate the virogenesis rates (and the extrinsic incubation periods) which corresponded to the different days of the year.

A *Culicoides* which emerged on day *x* and got infected *r* days after emergence *(TTCI= r)*, was assumed to complete a proportion (*eip _x+r_*) of the *EIP* on the day *x+r*:

$eip_{x+r}=1/EIP(T_{x+r})$

On the following day *(x+r+1)* the proportion of the *EIP* completed would be:

$eip_{x+r+1}=1/EIP(T_{x+r+1})$

The duration of the *EIP* for that *Culicoides* (*s*) would be given by the sum of the number of days needed so that the summatory of these proportions reaches one (i.e. the *EIP* is completed):

$\sum_{j=x+r}^{\infty} eip_{j}=1$

1.2.2. Time to the Next Blood Meal (*TNBM*)

The reciprocal of the biting rate (*b_r_*) is the interval between blood meals (*IBBM*), which for a given temperature *(T)* may be estimated as:

$IBBM(T)=1/b_{r}(T)$

Mean daily temperature data was used to calculate the biting rates (and the interval between blood meals) which corresponded to the different days of the year.

Once completed the *EIP*, in order to transmit the virus to a susceptible host, the vector has to take another blood meal. The time to the next blood meal *(TNBM)* for a given temperature *(T)* was modelled as:

*TNBM (T)* *= Uniform (0; IBBM (T))*

Therefore, the *Culicoides* which emerged on day *x*, got infected *r* days after emergence and needed *s* days to complete the *EIP* is assumed to complete a proportion (*tnbm _x+r+s_*) of the *TNBM* on the day *x+r+s*:

$tnbm_{x+r+s}=1/TNBM(T_{x+r+s})$

On the following day *(x+r+s+1)* the proportion of the *TNBM* completed would be:

$tnbm_{x+r+s+1}=1/TNBM(T_{x+r+s+1})$

The duration of the *TNBM* for that *Culicoides* (*t*) would be given by the sum of the number of days needed so that the summatory of these proportions reaches one (i.e. the *TNBM* is completed):

$\sum_{j=x+r+s}^{\infty} tnbm_{j}=1$

1.2.3. Probability of surviving the *EIP* and the *TNBM*

The probability of surviving the *EIP* and the *TNBM* (*P_EIP+TNBM_*) was obtained by calculating the proportion of *Culicoides* for which the longevity *(d)* is bigger than the sum of the *TTCI (r)* plus the *EIP (s)* plus the *TNBM (t)*:

$P_{EIP+TNBM}=P\left(d>\left[r+s+t\right]\right)$

***1.3. Probability of effective transmission***

Estimated independently for cattle and for sheep, taking into account:

1.3.1. The proportion of bites on cattle and on sheep

See section 1.1.1.1.

1.3.2. The proportion of cattle which were susceptible (*P_cS_*)

Calculated as one minus the proportion of the population immune. Immunity may have been achieved either after natural infection or by vaccination of the population against a specific serotype. As no vaccination was performed, the number of (naturally) immune cattle was estimated as the product of: number of cattle farms affected on the previous year, mean number of cattle per farm and mean within-farm prevalence (proportion of infected cattle within an infected farm). Then, the proportion of immune cattle was estimated by dividing the number of immune cattle by the cattle population. The proportion of sheep which were susceptible was also calculated. The values of the proportions of immune cattle and sheep for the German scenario are shown in Table 2.

1.3.3. The proportion of bites per infectious midge that infect a host (*β*)

In a given iteration, if *TTCI* (*r*) plus the *EIP* (*s*) plus the *TNBM* (*t*) was larger than the time from the emergence of the adult vector to the end of *PLVA*, overwintering occurred via the insect vector exclusively (pathway *I*) (Figure 1). If not, it is necessary the contribution of the host to reach the next season (overwintering in the insect vector and the host, i.e. pathway *III*), and for that an extra step is needed: the viraemia of the host needs to go beyond the end of *PLVA*.

***1.4. Probability the viraemia went beyond the end of the VFP***

When the time the virus spent on the host: time from infection to viraemia (*TITV*) plus duration of viraemia (*D_VC_*), was larger than the time from BTV transmission to the host to the end of the *PLVA*, the viraemia of the host went beyond the end of the *PLVA* (Figure 1).

***1.5. Overwintering by persistence of the virus in the ruminant host***

For overwintering to occur by persistence of the virus in cattle, the animal has to get infected before the *PLVA*, and then the viraemia has to last beyond the end of the *PLVA*.

Given infection of a cow the month before the *PLVA* (*month -1*), the probability of overwintering by persistence of BTV in cattle (*P_c-1_*) was calculated as:

$P_{c-1}=P\left(\left[DI+TIV_{c}+VP_{c}\right]>TEP_{m-1}\right)$

Where *DI* was the day (within that month) the cow got infected, *TIV_C_* the time from infection to viraemia in cattle, *VP_C_* the duration of viraemia in cattle and *TEP_m-1_* the time from start of *month -1* to the end of *PLVA* (Figure S2)*.*

The probability of overwintering by persistence of BTV in cattle for *month -2* (*P_c-2_*) was also calculated. Similarly, the probabilities of overwintering by persistence of BTV in sheep for *month -1* and *month -2* (*P_s-1_* and *P_s-2_*, respectively) were calculated.

**2. Sensitivity analysis**

For linear regression models, the assumptions of independence, normality and constant variance of the residuals of the model were checked. Independence of the residuals was assessed by means of the Durbin-Watson estimate. Values in the range of zero indicated that the assumption was satisfied. The normality assumption was assessed graphically by obtaining a histogram of standardized residuals and a normal probability plot. Similarly, constant variance of residuals was assessed by obtaining a scatter plot of the regression standardized residual versus the regression standardized predicted value, and checking that there was not a clear pattern. Besides, correlation among independent variables was assessed by obtaining the measure of tolerance. Values above 0.6 were considered as acceptable. For logistic regression models, the correlation between variable was assessed using the Pearson’s correlation coefficient. Values above 0.6 were considered as indicative of strong correlation.

**References**

[1] Gubbins S, Carpenter S, Baylis M, Wood JLN, Mellor PS: **Assessing the risk of bluetongue to UK livestock: uncertainty and sensitivity analyses of a temperature-dependent model for the basic reproduction number**. *J R Soc Interface* 2008, **5**:363-371.

[2] Gerry AC, Mullens BA: **Seasonal abundance and survivorship of *Culicoides sonorensis* (Diptera: Ceratopogonidae) at a southern California dairy, with reference to potential bluetongue virus transmission and persistence**. *J Med Entomol* 2000, **37**:675-688.

[3] EFSA: **Opinion of the Scientific Panel on Animal Health and Welfare on request from the Commission on bluetongue: Bluetongue vectors and insecticides**. *The EFSA Journal* 2008, **735**:1-70. http://www.efsa.europa.eu/en/scdocs/doc/ahaw_op_ej735_bluetongue2008_en,3.pdf.

[4] Mullens BA, Gerry AC, Lysyk TJ, Schmidtmann ET: **Environmental effects on vector competence and virogenesis of bluetongue virus in *Culicoides*: interpreting laboratory data in a field context**. *Vet Ital* 2004, **40**:160–166.

[5] Bonneau KR, DeMaula CD, Mullens BA, MacLachlan NJ : **Duration of viraemia infectious to *Culicoides sonorensis* in bluetongue virus-infected cattle and sheep**. *Vet Microbiol* 2002, **88**:115-125.

[6] Koumbati M, Mangana O, Nomikou K, Mellor PS, Papadopoulos O: **Duration of bluetongue viraemia and serological responses in experimentally infected European breeds of sheep and goats**. *Vet Microbiol* 1999, **64**:277-285.

[7] Cagienard A, Thür B, Griot C, Hamblin C, Stärk KDC: **No evidence of bluetongue virus in Switzerland**. *Vet Microbiol* 2006, **116**:13-20.

Figure S1: Diagram of the first 3 steps in the calculation of the probability of overwintering by pathways *I* & *III*.

Figure S2: Diagram of the calculation of the probability of overwintering by persistence of the virus in the ruminant host (pathway *II*).

Figure S1.


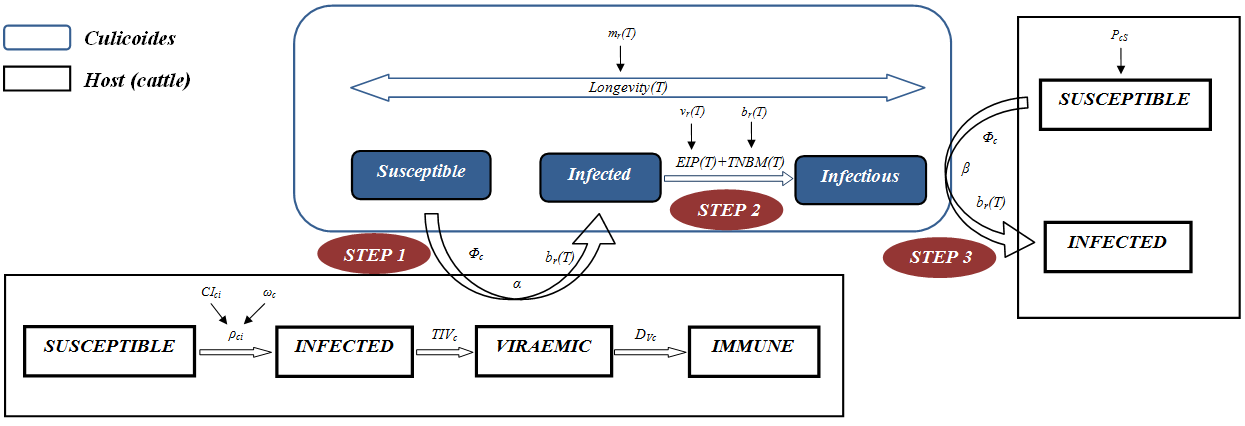

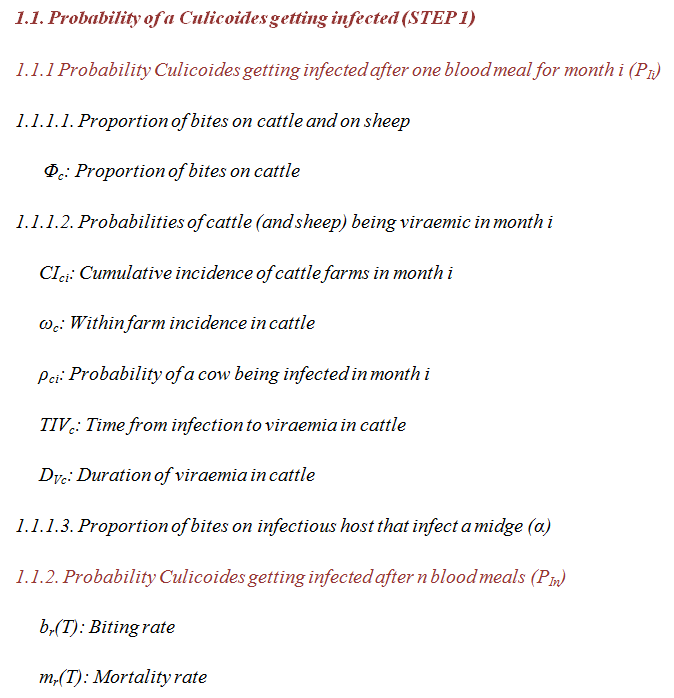


Figure S2.


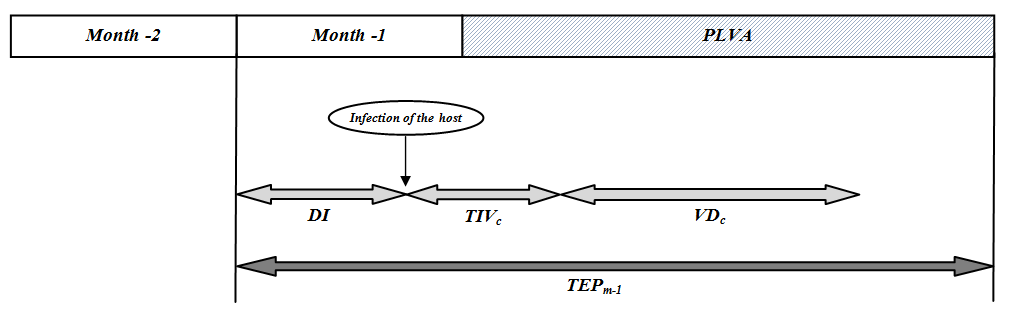


Table S1. Model input parameters: abbreviations, values, sources from which the values were obtained and section in which they are referred to in the supplementary material. (EOW: Expert Opinion Workshop).

| Input parameter | Abbreviation | Value | Source | Section |
| --- | --- | --- | --- | --- |
| Proportion of bites on cattle | *Φc_10_* | *Pert (0.19; 0.60; 0.81)* | EOW | 1.1.1.1. |
| Proportion of bites on sheep | *Φs_10_* | *1-[Pert (0.19; 0.60; 0.81)]* | EOW | 1.1.1.1. |
| Vector preference for cattle versus sheep | *σ* | *(1-Φc10)/ Φc10* | EOW | 1.1.1.1. |
| Day of infection within a month | *DI* | *Uniform (1;30)* |  | 1.1.1.2. & 1.5. |
| Time between infection and viraemia for cattle (days) | *TIV_c_* | *Uniform (7;14)* | [5] | 1.1.1.2. |
| Time between infection and viraemia for sheep (days) | *TIV_s_* | *Uniform (1;6)* | [6] | 1.1.1.2. |
| Duration of viraemia in cattle (days) | *D_Vc_* | *Gamma (5; 4.12)* | [1] | 1.1.1.2. |
| Duration of viraemia in sheep (days) | *D_Vs_* | *Gamma (14; 1.17)* | [1] | 1.1.1.2. |
| Mean probabilities of a cow being viraemic in month *i*,…, *i*+4 given infection in month *i* | *Various* | *p_vci_=0.20*  *p_vci+1_=0.48*  *p_vci+2_=0.02*  *p_vci+3_=1.4 x10^-4^*  *p_vci+4_=0.00* | Simulation | 1.1.1.2. |
| Days the cow remained viraemic in month *i* | *d_vci_* | *Various* | Simulation | 1.1.1.2. |
| Within farm incidence for cattle (and sheep) | *ω_ci_ (&ω_si_)* | *Pert (0.25; 0.40; 0.6)* | [7] | 1.1.1.2. |
| Mean probabilities of a sheep being viraemic in month *i*,…, *i*+3 given infection in month *i* | *Various* | *p_vsi_=0.38*  *p_vsi+1_=0.23*  *p_vsi+2_=5.8 x10^-5^*  *p_vsi+3_=0.00* | Simulation | 1.1.1.2. |
| Days the sheep remained viraemic in month *i* | *d_vsi_* | *Various* | Simulation | 1.1.1.2. |
| Proportion of bites on infectious host that infect a midge | *α* | *Uniform (0.001; 0.15)* | [1] | 1.1.1.3. |
| Mortality rate depending on the temperature (*T*) | *m_r_*(*T*) | $0.009\times\exp(0.16\times T)$ | [1, 2] | 1.1.2.1. |
| Biting rate depending on the temperature (*T*) | *b_r_*(*T*) | $0.00017\times T\times(T-3.7)\times(41.9-T)^{1/2.7}$ | [4] | 1.1.2.1. |
| Virogenesis rate depending on the temperature (*T*) | *v_r_*(*T*) | $0.0003\times T\times(T-10.41)$ | [4] | 1.2.1. |
| Proportion of bites per infectious midge that infect a host | *β* | *Uniform (0.8; 1)* | [1] | 1.3.3. |
| Time from start of *month -1* to the end of *PLVA* (in days) | *TEP_-1_* | *30+ PLVA* |  | 1.5. |

Table S2. Main model output parameters of the model: abbreviations, and section in which they are referred to in the supplementary material.

| Output parameter | Abbreviation | Section |
| --- | --- | --- |
| Probability of a *Culicoides* getting infected after one blood meal for month *i* | *P_Ii_* | 1.1.1. |
| Probability of a cattle being viraemic for month *i* | *P_Vci_* | 1.1.1.2. |
| Probability of a sheep being viraemic for month *i* | *P_Vsi_* | 1.1.1.2. |
| Probability of a *Culicoides* getting infected after *n* blood meals | *P_In_* | 1.1.2. |
| Daily probability of survival | *P_S_* | 1.1.2.1. |
| Day of the year *Culicoides* emerged (value obtained in a given iteration of the model) | *x* | 1.1.2.1. |
| Probability of a *Culicoides* surviving day *x* | *P_Sx_* | 1.1.2.1. |
| Probability of a *Culicoides* surviving just one day (given emergence on day *x*) | *P_Sx1_* | 1.1.2.1. |
| Longevity of *Culicoides* (in a given iteration of the model) | *d* | 1.1.2.1 |
| Daily probability the vector has taken a blood meal on day *x* | *P_BMx_* | 1.1.2.2. |
| Probability of a *Culicoides* getting infected on day *x* | *P_Ix_* | 1.1.2.2. |
| Time to *Culicoides* infection | *TTCI* | 1.1.2.3. |
| *TTCI* (value obtained in a given iteration of the model) | *r* | 1.2.1. |
| Extrinsic Incubation Period | *EIP* | 1.2.1. |
| Proportion of the *EIP* completed on day *x+r* | *eip _x+r_* | 1.2.1. |
| *EIP* (value obtained in a given iteration of the model) | *s* | 1.2.1. |
| Time to the Next Blood Meal | *TNBM* | 1.2.2. |
| Interval between blood meals | *IBBM* | 1.2.2. |
| Proportion of the *TNBM* completed on day *x+r+s* | *tnbm _x+r+s_* | 1.2.2. |
| *TNBM* (value obtained in a given iteration of the model) | *t* | 1.2.2. |
| Probability of surviving the *EIP* and the *TNBM* | *P_EIP+TNBM_* | 1.2.3. |
| Proportion of cattle which are susceptible | *P_cS_* | 1.3.2. |
| Probability of overwintering by persistence of BTV in cattle given infection of the cow the month before the start of the *PLVA* (month -1) | *P_c-1_* | 1.5 |

Table S3. Probabilities associated to the different days of survival used to construct the discrete distribution of *Culicoides* longevity.

| Longevity of *Culicoides* estimation | |
| --- | --- |
| Days of survival | Associated probability |
| *0* | $P_{Sx0}=(1-P_{Sx})$ |
| *1* | $P_{Sx1}=P_{Sx}\times(1-P_{Sx+1})$ |
| *2* | $P_{Sx2}=P_{Sx}\times P_{Sx+1}\times(1-P_{Sx+2})$ |
|  |  |
| *120* | $P_{Sx120}=P_{Sx}\times P_{Sx+1}\times....\times P_{Sx+119}\times(1-P_{Sx+120})$ |

Table S4: Probabilities associated to the different days to infection used to construct the discrete distribution of the time to *Culicoides* infection (*TTCI*).

| Time to *Culicoides* infection (*r*) estimation | |
| --- | --- |
| Days of infection (k) | Associated probabilities (p_k_) |
| *1* | *p_1_= (P_Ix_)/ (*$\sum_{j=0}^{d} P_{Ix+j}$*)* |
| *2* | *p_2_= (P_Ix+1_)/ ($\sum_{j=0}^{d} P_{Ix+j}$)* |
|  |  |
| *d* | *p_d_= (P_Ix+d_)/ ($\sum_{j=0}^{d} P_{Ix+j}$)* |
